# Conversion of *N‐*Acetylglucosamine to 3‐Acetamido‐5‐Acetylfuran over Al‐Exchanged Montmorillonite

**DOI:** 10.1002/open.202300148

**Published:** 2023-11-21

**Authors:** Kiyoyuki Yamazaki, Norihito Hiyoshi, Aritomo Yamaguchi

**Affiliations:** ^1^ Research Institute for Chemical Process Technology National Institute of Advanced Industrial Science and Technology (AIST) 4-2-1 Nigatake, Miyagino 983-8551 Sendai Japan

**Keywords:** biomass, clays, nitrogen-containing compounds, recyclable solid catalyst, shell biorefinery

## Abstract

3‐Acetamido‐5‐acetylfuran (3A5AF) is a potential platform compound for the production of nitrogen‐containing pharmaceuticals and chemicals. 3A5AF can be obtained by dehydration of chitin or its monomer, *N*‐acetylglucosamine (NAG). Here, we examined the use of solid catalysts for the dehydration of NAG to 3A5AF to achieve a more economical process that uses a recyclable catalyst. NAG was dehydrated using various solid catalysts in the presence of NaCl and *N,N*‐dimethyl acetamide as solvent at 433 K. The yield of 3A5AF with the solid catalysts decreased in the following order: Al‐exchanged montmorillonite>H‐ZSM‐5 (SiO_2_/Al_2_O_3_=40)>H‐montmorillonite (K‐10)>Amberlyst15>H‐ZSM‐5 (SiO_2_/Al_2_O_3_=300)>TiO_2_>γ‐Al_2_O_3_>ZrO_2_>SiO_2_ ⋅ MgO>Na‐montmorillonite. The highest yield of 3A5AF (14 %) was obtained with the Al‐exchanged montmorillonite. The montmorillonite catalysts were characterized by using inductively coupled plasma optical emission spectroscopy, energy‐dispersive X‐ray spectroscopy, N_2_ adsorption, Fourier‐transformed infrared spectroscopy, X‐ray diffraction, and ^27^Al magic‐angle spinning nuclear magnetic resonance spectroscopy (MAS‐NMR). In addition, a combined catalyst of Al‐exchanged montmorillonite and Cl^−^ from synthetic hydrotalcite was found to be an active and recyclable solid catalyst for NAG dehydration to 3A5AF.

## Graphical abstract

Dehydration of *N*‐acetylglucosamine (NAG) to 3‐acetamido‐5‐acetylfuran (3A5AF) over solid catalysts was performed, and the use of chloride ion from a heterogeneous catalyst instead of from sodium chloride was investigated. Based on our findings, we propose a recyclable solid catalyst system that uses Al‐exchanged montmorillonite and Cl‐containing synthetic hydrotalcite for NAG dehydration to 3A5AF.

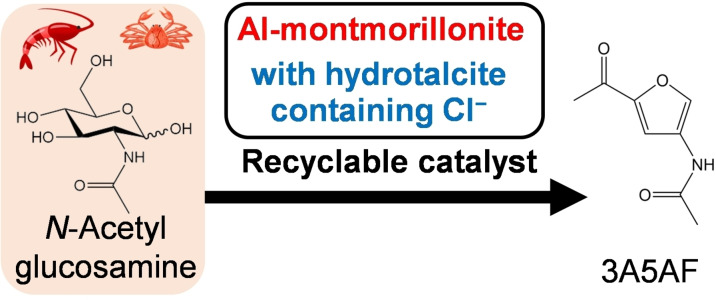

## Introduction

**Scheme 1:**
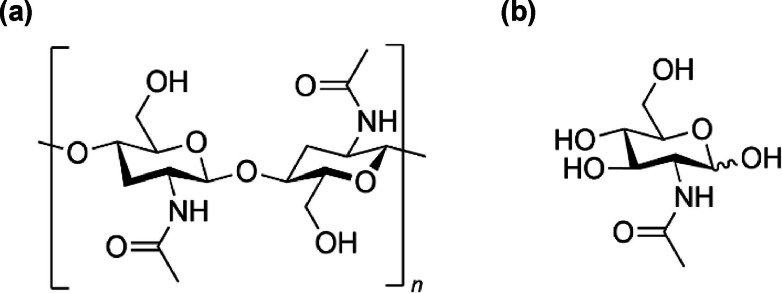
Chemical structure of (a) chitin and (b) *N*‐acetylglucosamine (NAG).

Valorization of biomass into value‐added chemicals is expected to contribute to a sustainable society.^[1]^ Chitin, a biopolymer of *N‐*acetylglucosamine (NAG) bound by β‐1,4‐glycosidic bonds that is found in crustacean shells, is the second most abundant biomass resource after cellulose and a promising resource for the production of chemicals.^[1]^ Although the molecular structures of chitin and cellulose are similar^[2]^ (Scheme 1), chitin contains nitrogen‐containing groups (acetamido group), whereas cellulose does not. Recently, conversions of chitin into high‐value, nitrogen‐containing chemicals such as pharmaceuticals, raw materials for the production of aromatic polymers, amide alcohols, and amino acid derivatives have attracted much attention.^[3]^

**Scheme 2:**
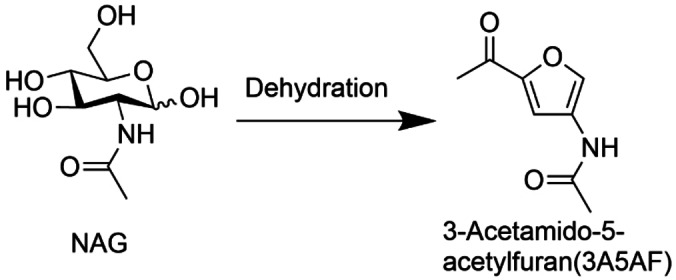
Dehydration of NAG to 3A5AF.

3‐Acetamido‐5‐acetylfuran (3A5AF) is a potential platform chemical obtained from chitin for the production of high‐value, nitrogen‐containing pharmaceuticals and chemicals such as anti‐cancer agents^[3b]^ and amino cyclopentenone derivatives.^[4]^ 3A5AF has been found among the products of pyrolysis of chitin (max. yield: 2 %).^[5]^ To overcome this low yield, several homogeneous catalysts for the NAG dehydration to 3A5AF (Scheme 2) have recently been proposed. Omari et al. obtained 3A5AF (yield: 58 %) by dehydrating NAG with boric acid and NaCl at 493 K with a microwave used for rapid heating.^[6]^ Wang et al. obtained 3A5AF (yield: 43 %) by dehydrating NAG at 473 K with an ionic liquid and glycine chloride.^[7]^ Chen et al. also obtained 3A5AF (yield: 56 %) by dehydrating NAG with an ionic liquid, with the highest yield being obtained with 3‐methylimidazolium chloride, boric acid, and NaCl at 453 K.^[8a]^ Padovan et al. reported NAG dehydration to 3A5AF (yield: 30 %) using only aluminum chloride as the catalyst at a lower temperature (393 K).^[8b]^ Although there are some reports using homogeneous catalysts, there are few reports on the conversion reaction of NAG to 3A5AF using heterogeneous catalysts. Shaikh et al. reported that lanthanum oxide catalyzed the conversion of NAG to 3A5AF as a heterogeneous catalyst.^[9]^ 3A5AF formation from NAG proceeds via ring opening of NAG, formation of a five‐membered ring, and dehydration.^[7]^ Chloride ion catalyzes the ring opening of NAG and formation of the five‐membered ring,^[7]^ as well as the subsequent dehydration.^[10]^ However, although it is possible to obtain 3A5AF by using chloride ion and acid catalysts, the processes reported to date require large amounts of catalysts (3–4 times the amount of NAG),[^6^, ^7^, ^8^] and it is difficult to recover the spent catalyst for recycling by extraction from reaction solution.

Here, we explored the use of solid catalysts for NAG dehydration to 3A5AF with the goal of developing a more economical process that uses a recyclable catalyst. To this end, we performed NAG dehydration over various solid acid catalysts in the presence of chloride ion and identified Al‐exchanged montmorillonite as a promising catalyst. We also investigated the use of chloride ion from heterogeneous catalysts instead of from sodium chloride. Based on our present findings, we propose a solid catalyst system using Al‐exchanged montmorillonite and a synthetic hydrotalcite containing Cl^−^ for the dehydration of NAG to 3A5AF.

## Results and Discussion

### Screening of solid catalysts

Dehydration of NAG was performed in *N,N*‐dimethyl acetamide (DMA) with NaCl and various solid acid catalysts (H‐ZSM‐5 [SiO_2_/Al_2_O_3_=40 or 300], Amberlyst15, cation‐exchanged montmorillonites [K‐10 or Al‐Mont], and several metal oxides [ZrO_2_, γ‐Al_2_O_3_, TiO_2_, or SiO_2_ ⋅ MgO]) at 433 K for 2 h. Table 1 shows the conversions of NAG and product yields obtained. Among the catalysts examined, Al(4.0)‐Mont and Al(0.5)‐Mont afforded the greatest yields of 3A5AF (both 14 %). The yields of 3A5AF decreased in the following order: Al(4.0)‐Mont=Al(0.5)‐Mont>H‐ZSM‐5 (SiO_2_/Al_2_O_3_=40)>K‐10>Amberlyst15>H‐ZSM‐5 (SiO_2_/Al_2_O_3_=300)>TiO_2_>γ‐Al_2_O_3_>ZrO_2_>SiO_2_ ⋅ MgO>Na‐Mont.

**Table 1:** NAG conversions over various solid catalysts.^[a]^

Catalyst	Amount of catalyst (g)	NAG conversion (%)	Yield (%)		
			3A5AF	HMF	AEF
None	0	93	0.9	0.0	0.2
Na‐Mont	2.0	97	0.0	0.0	0.0
Al(0.5)‐Mont	2.0	96	14	13	0.7
Al(4.0)‐Mont	2.0	99	14	15	0.6
K‐10	2.0	96	8.7	9.6	0.2
MFI(SiO_2_/Al_2_O_3_=40)	2.0	83	9.3	20	0.0
MFI(SiO_2_/Al_2_O_3_=300)	2.0	72	4.6	0.0	0.1
Amberlyst15	2.0	100	9.3	0.0	1.5
SiO_2_⋅MgO	2.0	100	1.3	0.0	0.0
ZrO_2_	0.5	94	2.2	0.0	0.0
γ‐Al_2_O_3_	0.5	94	2.0	0.0	0.0
TiO_2_	0.5	81	3.0	0.0	0.1

[a] NAG, 6.8 mmol; DMA, 30 mL; NaCl, 2 mmol; reaction time, 2 h; reaction temperature, 433 K.

5‐Hydroxymethylfurfural (HMF) was also produced from NAG when the catalyst was H‐ZSM‐5 or Al‐Mont (Table 1). HMF is a furan compound obtained from the dehydration of glucose.^[10]^ Glucose was also detected as a by‐product of NAG conversion over Al(4.0)‐Mont, although the amount could not be quantified because peaks derived from glucose and other products could not be separated well in the chromatograph. Thus, NAG might be converted to glucose via deacetylation and deamination and then dehydrated to HMF. In addition, another N‐containing compound, 3‐acetamido‐5‐ethylidene furanone (AEF),^[8b]^ was also formed over several of the catalysts, albeit at very low yields (<1.3 %). Also, the polymerization reaction might occur, resulting in the formation of humin.

Our findings indicated that Al‐exchanged montmorillonite is a promising catalyst for the synthesis of 3A5AF from NAG. When Na‐Mont was used instead of Al‐Mont, 3A5AF was not formed. Thus, the active sites for 3A5AF formation must have been created by the Al‐exchange treatment of Na‐Mont. Montmorillonite is an aluminosilicate composed of layers of SiO_4_ tetrahedra and AlO_6_ octahedra.^[11]^ Some of the Si^4+^ in the SiO_4_ tetrahedra and Al^3+^ in the AlO_6_ octahedra are substituted with Al^3+^ and Mg^2+^, respectively, resulting in aluminosilicate layers with a negative charge. Na^+^ cations in the interlayer spaces, which are exchangeable with other cations, maintain a neutral charge. Cation exchange of montmorillonites is reported to produce both Brønsted and Lewis acid sites.^[12]^ Therefore, it is probable that Brønsted and/or Lewis acid sites are formed in Al(4.0)‐Mont and Al(0.5)‐Mont, and it is these sites that catalyze dehydration of NAG to 3A5AF.

### Characterization of montmorillonite catalysts

Table 2 shows the results of the elemental analysis of the montmorillonite catalysts. ICP analysis of the solutions after ion exchange revealed that most of the Na^+^ cations in the Na‐Mont were released into solution during the ion‐exchange treatment and that Al(0.5)‐Mont and Al(4.0)‐Mont did not contain Na^+^ cations. For Al(0.5)‐Mont, the uptake of Al^3+^ (0.4 mmol ⋅ g^−1^) was about one‐third of the amount of Na^+^ released (1.5 mmol ⋅ g^−1^), indicating that the Na^+^−Al^3+^ exchange proceeded completely. In contrast, no uptake of Al^3+^ was detected in Al(4.0)‐Mont. One possibility for this is that leaching of Al^3+^ from the aluminosilicate layer occurred during the ion‐exchange treatment. It is known that collapse of the aluminosilicate layer of montmorillonite and dealumination occur in acidic solutions.^[13]^ In the present ion‐exchange treatment, the pH of the solution used for Al(4.0)‐Mont (pH=2.4) was lower than that used for Al(0.5)‐Mont (pH=3.2), suggesting that leaching of Al^3+^ from the aluminosilicate layer of Al(4.0)‐Mont did indeed occur. NMR analysis revealed that Al^3+^ cations were present at ion‐exchange sites in Al(4.0)‐Mont. Thus, it is probable that leaching of Al^3+^ and Na^+^−Al^3+^ exchange proceeded simultaneously in the ion‐exchange treatment of Al(4.0)‐Mont.

**Table 2:** Composition of montmorillonite catalysts.

Catalyst	Si/Al^[a]^ (mol/mol)	Composition^[a]^ (wt %)						Na^+^ released^[b]^ (mmol/g)	Al^3+^ uptake^[b]^ (mmol/g)
		Na_2_O	SiO_2_	Al_2_O_3_	MgO	Fe_2_O_3_	CaO		
Na‐Mont	2.3	4.5	51.6	19.3	2.8	2.1	1.9	–	–
Al(0.5)‐Mont	2.1	0.3	53.6	21.8	3.2	2.1	1.0	1.5	0.4
Al(4.0)‐Mont	2.3	0.9	55.4	20.2	3.0	2.2.	0.5	1.6	0.0
K‐10	4.5	0.7	64.5	12.0	1.7	2.6	0.6	–	–

[a] Measured by STEM‐EDS; [b] amount of Na^+^ released and that of Al^3+^ uptake in the ion‐exchange step was measured by ICP analysis of the reaction solution after ion‐exchange.

**Figure 1:**
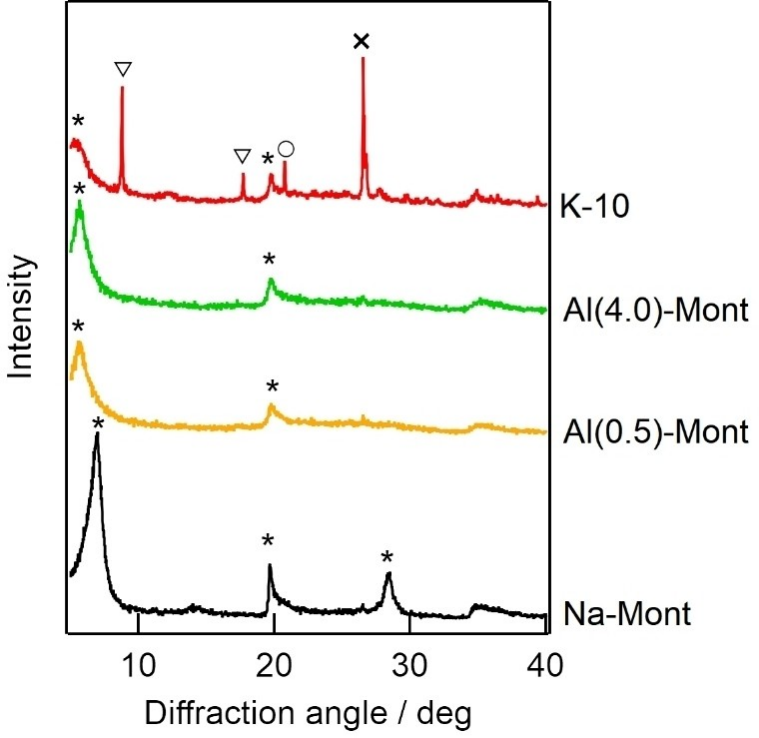
XRD patterns of montmorillonites. Montmorillonite (*), muscovite (▿), tridymite (○), and quartz (×).

Figure 1 shows the XRD patterns of the montmorillonite catalysts. In the XRD patterns of Al(0.5)‐Mont and Al(4.0)‐Mont, all peaks observed are attributable to the montmorillonite phase. The peaks of the montmorillonite phase in the XRD patterns of Al(0.5)‐Mont and Al(4.0)‐Mont are broader than those of Na‐Mont, indicating that the crystallinity was lowered during the ion‐exchange treatment. In the XRD pattern of K‐10, peaks attributable to other minerals were also observed in addition to the montmorillonite phase. The peaks of the montmorillonite phase in the XRD pattern of K‐10 were the broadest among those of the montmorillonite catalysts. Furthermore, crystalline SiO_2_ (quartz and tridymite) and muscovite were detected as impurity phases.^[14]^ The aluminosilicate layer in K‐10 is partially collapsed and transformed to the SiO_2_ phase because H‐montmorillonite K‐10 is produced using strong acid treatments.[^12b^, ^14b^, ^15^] The broad peaks of the montmorillonite phase in the XRD patterns of K‐10 indicated partial collapse of the aluminosilicate layer. Table 3 shows the surface area of the montmorillonite catalysts. The surface area decreased in the following order: K‐10>Al(4.0)‐Mont>Al(0.5)‐Mont>Na‐Mont. It is probable that the surface area increased as more of the aluminosilicate layers collapsed.

**Table 3:** Specific surface area of the catalysts.

Montmorillonite	Specific surface area^[a]^ (m^2^ g^−1^)
Na‐Mont	31
Al(0.5)‐Mont	97
Al(4.0)‐Mont	120
K‐10	200

[a] Measured by N_2_ adsorption.

**Figure 2:**
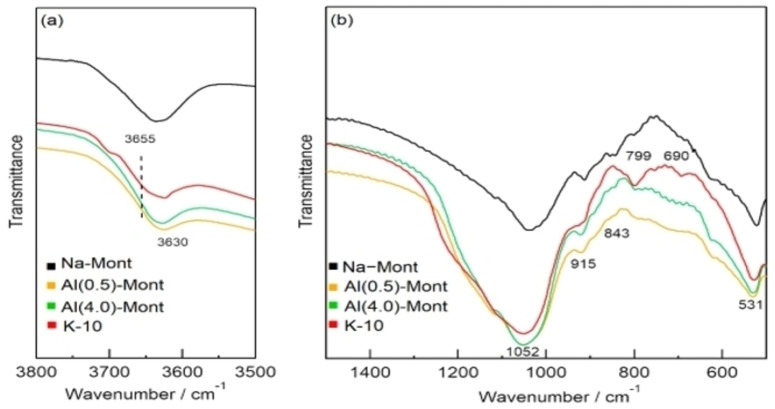
FT‐IR spectra of montmorillonite catalysts. (a) 3800–3500 cm^−1^, (b) 1500–500 cm^−1^.

Figure 2 shows the FT‐IR spectra of the montmorillonite catalysts. The assignments of the absorption bands are summarized in Table 4.[^11b^, ^16^] In the FT‐IR spectrum of K‐10, absorption bands at 799 and 690 cm^−1^ ascribed to tridymite and quartz, respectively, were observed. This result was in agreement with the findings of the XRD analysis. In the FT‐IR spectra of Al(4.0)‐Mont and Al(0.5)‐Mont, broad bands around 799 and 690 cm^−1^ were also observed, suggesting that the aluminosilicate layers of montmorillonite were partially collapsed and transformed to the SiO_2_ phase. Tyagi et al. have reported that the absorption band of the O−H vibration of a Brønsted acid is present at 3655 cm^−1^.^[11b]^ In the FT‐IR spectra of Al(4.0)‐Mont and Al(0.5)‐Mont, no absorption band at 3655 cm^−1^ was observed. In contrast, a broad absorption band at 3655 cm^−1^ was observed in the FT‐IR spectrum of K‐10. These results indicated that Al‐exchanged montmorillonite did not have Brønsted acid sites. Conversely, K‐10 had Brønsted acid sites.

**Table 4:** FT‐IR band assignments for montmorillonite.[^11b^, ^16^]

Wavenumber (cm^−1^)	Assignment
3655	Brønsted acid site
3630	SiO−H stretching
1052	Si−O stretching
915	Al (OH)Al bending
843	Al (OH)Mg bending
799	Tridymite
690	Quartz
531	Si−O bending

**Figure 3:**
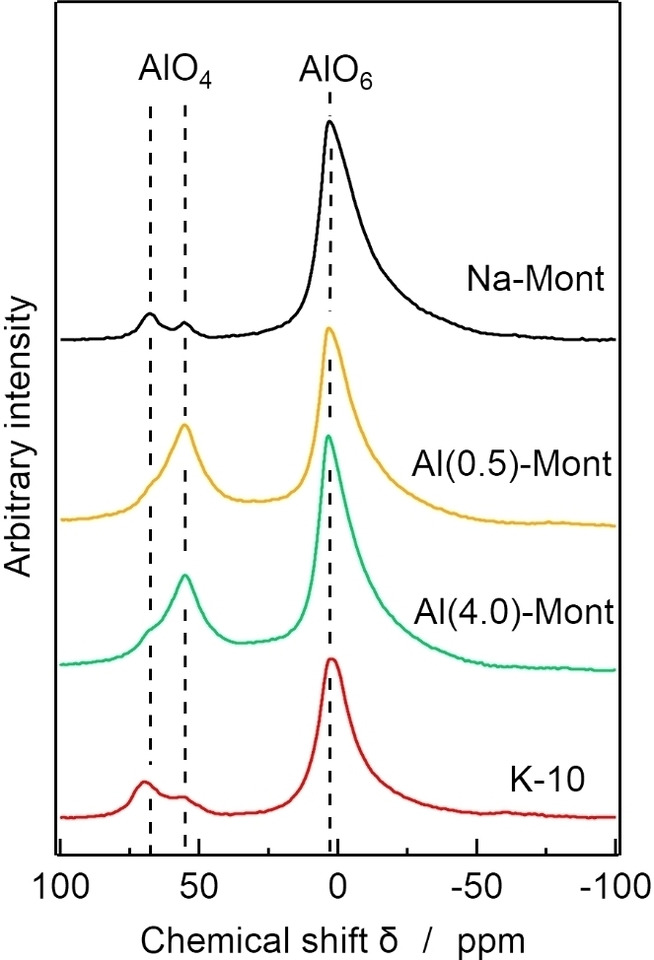
^27^Al magic‐angle spinning NMR spectra of montmorillonite catalysts.

Figure 3 shows ^27^Al MAS NMR spectra of the montmorillonite catalysts. Chemical shifts at 3.2, 55, and 68 ppm were observed in the NMR spectra of Na‐Mont, Al(0.5)‐Mont, Al(4.0)‐Mont, and K‐10. The chemical shift at 3.2 ppm is attributed to Al^3+^ in the AlO_6_ octahedra of the aluminosilicate layer.^[17]^ The chemical shifts at 55 and 68 ppm are attributed to Al^3+^ in the AlO_4_ tetrahedra.^[17]^

The NMR spectrum of Al(0.5)‐Mont shows a larger peak at 55 ppm than that of Na‐Mont. Therefore, the chemical shift at 55 ppm is attributed to Al^3+^ in ion‐exchange sites. The chemical shift at 68 ppm is attributed to Al^3+^ substituted for Si^4+^ in SiO_4_ tetrahedra of the aluminosilicate layer. The NMR spectrum of Al(4.0)‐Mont also showed a larger peak at 55 ppm than that of Na‐Mont, indicating that Al(4.0)‐Mont contained exchanged Al^3+^ cations. It should be noted that the peaks at 55 ppm were much larger in the spectra of Al(0.5)‐Mont and Al(4.0)‐Mont than in the spectrum of K‐10. This indicates of Al^3+^ at their ion‐exchange sites. We attributed the higher yield of 3A5AF obtained with Al(0.5)‐Mont and Al(4.0)‐Mont compared with that obtained with K‐10 to the larger amount of Al^3+^ cations at ion‐exchange sites that work as the active sites for 3A5AF formation, although further studies are needed to elucidate the detailed structure of these active sites.

### Optimization of reaction conditions

**Figure 4:**
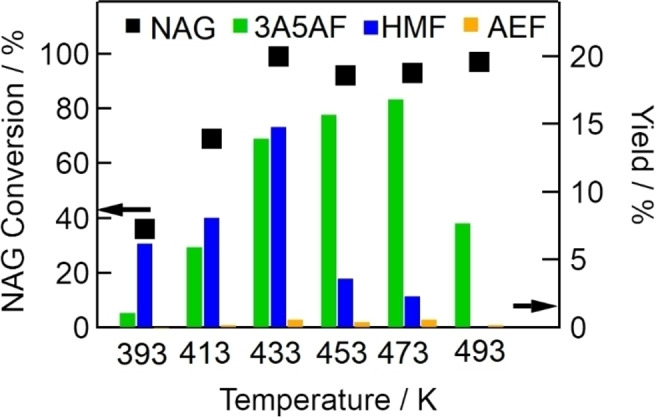
Effects of reaction temperature (393–493 K) on NAG conversion (dots, left axis) and yields of 3A5AF, HMF, and AEF (bars, right axis) (NAG, 6.8 mmol; DMA, 30 mL; Al(4.0)‐Mont, 2.0 g; NaCl, 2 mmol; reaction time, 2 h).

We conducted several experiments to optimize the reaction conditions for NAG dehydration to 3A5AF over Al(4.0)‐Mont. Figure 4 shows the conversion of NAG and yields of 3A5AF, HMF, and AEF at various temperatures over Al(4.0)‐Mont with NaCl.

The yields of 3A5AF increased to 17 % with increasing reaction temperature up to 473 K, and thereafter decreased to 7.7 % at 493 K. The yield of HMF also increased to 15 % with increasing reaction temperature up to 433 K; however, the yield of HMF decreased with increasing temperature in the temperature range 433–493 K. These findings suggest that 3A5AF and HMF are decomposed at temperatures above 493 K and 453 K, respectively.

**Figure 5:**
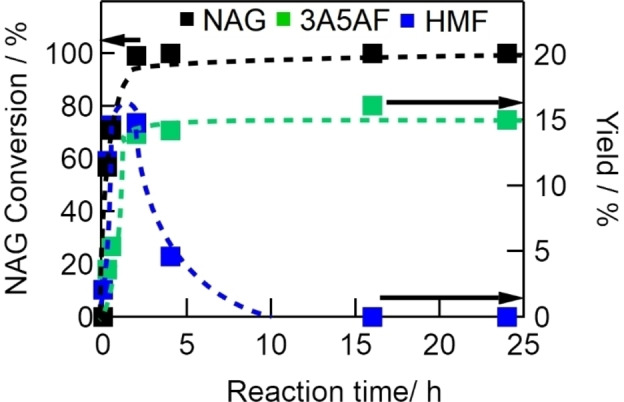
Effects of reaction time (0–24 h) on NAG conversion (left axis) and yields of 3A5AF and HMF (right axis) (NAG, 6.8 mmol; DMA, 30 mL; Al(4.0)‐Mont, 2.0 g; NaCl, 2 mmol; reaction temperature, 433 K).

Figure 5 shows the reaction profile of conversion of NAG at 433 K out to a reaction time of 24 h. NAG conversion reached almost 100 % at 2 h. The yield of 3A5AF increased up to 14 % at 2 h and remained constant thereafter. Although the yield of HMF also increased up to 2 h, it rapidly decreased to zero with increasing reaction time. The implications here are that 3A5AF is stable at 433 K and resists further conversion.

**Figure 6:**
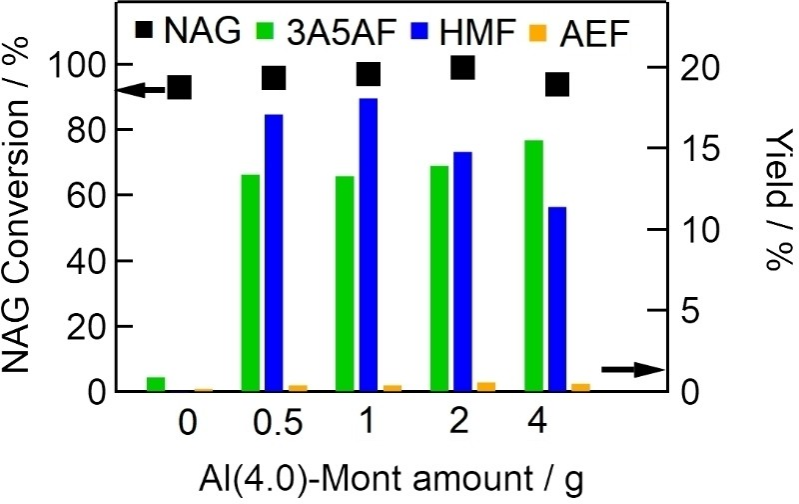
Effects of catalyst amount (Al(4.0)‐Mont: 0–4 g) on NAG conversion (dots, left axis) and yields of 3A5AF, HMF, and AEF (bars, right axis) (NAG, 6.8 mmol; DMA, 30 mL; NaCl, 2 mmol; reaction temperature, 433 K; reaction time, 2 h).

Figure 6 shows the conversion of NAG and yields of 3A5AF, HMF, and AEF at 433 K at 2 h with different amounts of Al(4.0)‐Mont catalyst (0–4 g). NAG conversion remained at almost 100 % irrespective of the amount of catalyst used. The yield of 3A5AF was 13 % with 0.5 g Al(4.0)‐Mont and increased slightly with increasing amount of catalyst. The yield of HMF increased at catalyst amounts up to 1 g, but decreased at amounts of 2 g or more. These findings indicate that conversion of HMF to smaller molecules was increased with increasing amount of catalyst.

### Catalyst recyclability

**Figure 7:**
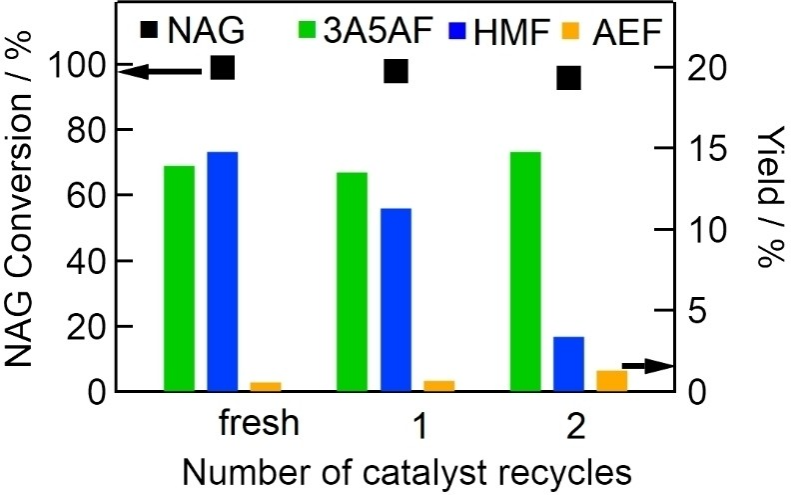
Effects of catalyst recycling on NAG conversion (dots, left axis) and yields of 3A5AF, HMF, and AEF (bars, right axis) (NAG, 6.8 mmol; DMA, 30 mL; Al (4.0)‐Mont, 2.0 g; reaction temperature, 433 K; reaction time, 2 h).

To examine catalyst deactivation during the reaction, we performed a recycling test using Al(4.0)‐Mont for NAG dehydration with NaCl. Figure 7 shows the conversion of NAG and yields of 3A5AF, HMF, and AEF obtained with fresh or recycled catalyst. Although the yield of 3A5AF was maintained even with recycled Al(4.0)‐Mont, the yield of HMF gradually decreased with increasing reuse cycles. It is possible that Al‐exchanged montmorillonite has different active sites for HMF formation and 3A5AF formation. If deactivation of active sites for HMF formation occurs while active sites for 3A5AF formation remain active after the reaction, the decrease of HMF yields can be explained.

Although Al(4.0)‐Mont was found to be a recyclable catalyst for NAG dehydration to 3A5AF, the process required NaCl every time. We therefore wanted to develop a recyclable solid catalyst for NAG dehydration to 3A5AF that did not need NaCl.

**Figure 8:**
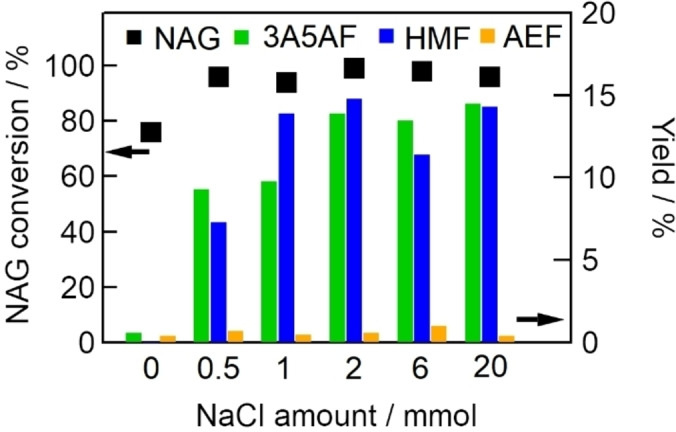
Effects of NaCl amount (0–20 mmol) on NAG conversion (dots, left axis) and yields of 3A5AF, HMF, and AEF (bars, right axis) (NAG, 6.8 mmol; DMA, 30 mL; Al(4.0)‐Mont, 2.0 g; reaction temperature, 433 K; reaction time, 2 h).

We considered the effects of NaCl (0–20 mmol) on NAG dehydration to 3A5AF. Figure 8 shows the conversion of NAG and yields of 3A5AF, HMF, and AEF with various amounts of NaCl (0–20 mmol). NAG conversion was almost 100 % with 0.5 mmol NaCl, and the conversion remained at that level when the amount of NaCl was further increased. The yields of 3A5AF and HMF increased with increasing amounts of NaCl up to 2 mmol and remained at those levels when the amount of NaCl was further increased.

Table 5 shows the conversion of NAG and yields of 3A5AF AEF obtained over Al(4.0)‐Mont in the presence of various additives instead of NaCl. The 3A5AF yields decreased in the order of Cl^−^>Br^−^>F^−^. One reason why little 3A5AF was obtained with fluoride salt could be that fluoride, but not chloride or bromide, was hydrolyzed by water derived from the dehydration of NAG, resulting in the formation of weak basicity. In NAG dehydration, chloride or bromide ion would catalyze the ring opening of NAG and formation of the five‐membered ring. The counter cations of halide (Na^+^, K^+^, TBA^+^) had little effect on the yield of 3A5AF.

**Table 5:** NAG conversion and yields of 3A5AF and AEF obtained over Al(4.0)‐Mont in the presence of various additives (NAG, 6.8 mmol; DMA, 30 mL; Al(4.0)‐Mont, 2.0 g; additives 2 mmol; reaction temperature, 433 K; reaction time 2 h).

Additive	NAG conversion (%)	Yield (%)		
		3A5AF	HMF	AEF
None	49	0.0	0.0	0.0
NaF	88	0.0	0.4	0.2
KF	91	1.4	0,0	0.5
NaCl	99	14	15	0.6
KCl	89	13	7.8	1.0
TBACl	95	12	10	0.7
NaBr	91	3.3	13	0.7
KBr	100	3.1	9.1	0.9
TBABr	90	2.8	8.3	1.9

**Figure 9:**
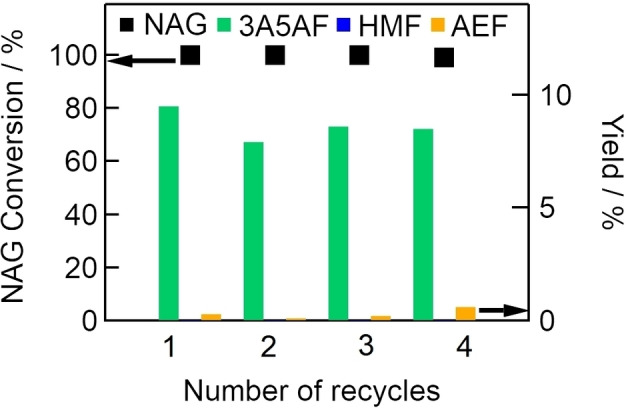
Effects of combined Al(4.0)‐Mont and Cl‐HT catalyst on NAG conversion (left axis) and yields of 3A5AF and AEF (right axis) over several reuse cycles (NAG, 6.8 mmol; DMA, 30 mL; Al(4.0)‐Mont, 1.0 g; Cl‐HT, 1.0 g; reaction temperature, 473 K).

Since Cl^−^ was found to effectively catalyze NAG dehydration to 3A5AF, we examined the use of synthetic hydrotalcite containing Cl^−^ (Cl‐HT) as a recyclable solid catalyst for NAG dehydration to 3A5AF. Figure 9 shows the conversion of NAG and yields of 3A5AF and AEF obtained in a recycling test using a combined catalyst of Al(4.0)‐Mont and Cl‐HT at 473 K without NaCl. The combined catalyst was able to promote NAG dehydration to 3A5AF without NaCl though the yield of 3A5AF was slightly lower than that obtained with Al‐Mont and NaCl (17 % at 473 K shown in Figure 4). We considered that HMF would not be observed because of high reaction temperature (473 K). In addition, Figure 9 also shows that the yield of 3A5AF was maintained at about 9 % at the fourth use of the combined catalyst. This result suggests that the combined catalyst was a suitable recyclable solid catalyst for NAG conversion to 3A5AF without NaCl.

## Conclusions

Here, we examined the use of solid catalysts, including zeolite, cation exchange resin, cation‐exchanged montmorillonite, and metal oxides, for the conversion of NAG to 3A5AF. Among them, Al‐exchanged montmorillonite was the best catalyst for NAG dehydration to 3A5AF. The Al‐exchange treatment of Na‐Mont increased exchanged Al^3+^ cations with fourfold coordination. The fourfold‐coordinated Al^3+^ cations were active for the formation of 3A5AF. That explains why Al‐exchanged montmorillonite the most active catalyst for the formation of 3A5AF in this study was. We also examined NAG dehydration to 3A5AF over a combined catalyst of Al‐exchanged montmorillonite and synthetic hydrotalcite containing Cl^−^. Several homogeneous catalysts have been previously proposed,[^11^, ^12^, ^13^, ^14^] some of which are recyclable;^[12]^ however, the recovery of the homogeneous catalysts is a laborious process. We found that our combined catalyst was an active solid catalyst that could be recovered by filtering only, with the yield 3A5AF remaining at about 9 % at the fourth use of the catalyst. In the future, we intend to optimize the Al‐exchange conditions to increase the number tetrahedral Al sites and improve the yield of 3A5AF obtained from the conversion of NAG.

## Experimental Section

**Materials**. *N‐*Acetylglucosamine (NAG), *N*,*N*‐dimethyl acetamide (DMA), sodium chloride (NaCl), potassium chloride (KCl), tetrabutylammonium chloride (TBACl), sodium bromide (NaBr), potassium bromide (KBr), tetrabutylammonium bromide (TBABr), sodium fluoride (NaF), potassium fluoride (KF), aluminum nitrate nonahydrate (Al(NO_3_)_3_ ⋅ 9H_2_O), ethanol, and cation‐exchange resin (Amberlyst15) were purchased from FUJIFILM Wako Pure Chemical Corporation. Silica‐magnesia (SiO_2_ ⋅ MgO, JRC‐SM‐2, Mizusawa Industrial Chemicals, Ltd.) and zirconia (ZrO_2_, JRC‐ZRO‐7, Daiichi Kigenso Kagaku Kogyo Co., Ltd.) were obtained from the Catalysis Society of Japan. Alumina (γ‐Al_2_O_3_, AKP‐G015) was purchased from Sumitomo Chemical Co., Ltd. Titania (TiO_2_, P‐25) was purchased from Nippon Aerosil Co., Ltd. H‐ZSM‐5 zeolite catalysts (MFI, SiO_2_/Al_2_O_3_=40 or 300) were purchased from Nikki‐Universal Co., Ltd. Na‐type montmorillonite (Kunipia‐F, denoted as Na‐Mont) was purchased from Kunimine Industries Co., Ltd. H‐type montmorillonite (K‐10) was purchased from Merck & Co., Inc. Synthetic hydrotalcite containing Cl^−^ (MA‐CL, denoted as Cl‐HT) was purchased from Kyoeisha Chemical Co., Ltd. Composition of Cl‐HT is [Mg_0.9_Al_0.1_(OH)_2_][Cl_0.1_ ⋅ 0.5H_2_O]. Chloride ion was included by 1 mmol in montmorillonite per gram.

**Preparation of Al‐exchanged montmorillonite**. Aluminum‐exchange of Na‐Mont was performed in accordance with the literature.^[18]^ In short, Na‐Mont (20 g), Al(NO_3_)_3_ ⋅ 9H_2_O (3.6 or 30 g; 0.5 and 4.0 mmol‐Al/g‐montmorillonite, respectively), and water (160 g) were added to an eggplant flask and stirred for 2 h. After stirring, the solid was collected by filtration and washed with water and aqueous ethanol. The obtained solid was dried at 333 K in an oven, crushed with a ball mill, and sifted with a sieve (less than 0.26 mm). Hereafter, the aluminum‐exchanged montmorillonite samples are denoted as Al(0.5)‐Mont and Al(4.0)‐Mont depending on the initial Al/montmorillonite ratio.

**Dehydration of** ***N*** **‐acetylglucosamine**. Dehydration of NAG was performed in a stainless‐steel batch reactor (inner volume: 100 cm^3^) equipped with a screw mixer (MMJ‐100, O‐M Lab Tech Co., Ltd.). NAG (1.5 g, 6.8 mmol). A solid catalyst (2.0 g), NaCl (0.12 g, 2.0 mmol) as an additive, and DMA (30 cm^3^) were put into the reactor. Then, the reactor was heated to 433 K and kept at the reaction temperature for 2 h. At the end of the reaction, the reactor was cooled to ambient temperature, and the solution was filtered using a membrane filter (the pore size: 0.2 μm) to remove the spent solid catalyst.

Quantitative analyses of NAG, 3A5AF, 5‐hydroxymethylfurfural (HMF), and 3‐acetamido‐5‐ethylidene furanone (AEF) were performed by using a high‐performance liquid chromatograph (Shimadzu) equipped with a refractive index detector (Shimadzu, RID‐10A), a UV–vis detector (Shimadzu, SPD‐ 20AV), and a Rezex RPM‐Monosaccharide Pb^2+^ column (Phenomenex). Conversion of NAG and the yield of each product were calculated as follows:

(1) ConversionofNAG(%)={(initialamountofNAG(mmol))-(amountofNAGafterreaction(mmol))/(amountofinitialNAG(mmol))×100

(2) Productyield(%)=(amountofproduct(mmol))/(initialamountNAG(mmol))×100

As a catalyst recycling test, spent catalyst was recovered by filtering and rinsing with water (about 30 g). After drying overnight at 333 K in an oven, the recovered catalyst was used for the next reaction experiment.

**Characterization**. To calculate the amount of exchanged Al^3+^ and Na^+^ in the montmorillonite samples, the concentrations of Na^+^ and Al^3+^ ions in the solution before and after Al‐exchange of Na‐Mont were measured by inductively coupled plasma optical emission spectroscopy (ICP‐OES) using an SPS3100 spectrometer (SII Nanotechnology Inc.). The X‐ray diffraction (XRD) patterns of the catalysts were recorded on a Rigaku SmartLab instrument operating at a current of 30 mA, a voltage of 40 kV, and a 2θ step size of 0.02°; Cu Kα radiation was used. The Fourier‐transform infrared (FT‐IR) spectra of the montmorillonite catalysts were collected on a Frontier system (PerkinElmer) equipped with a DTG detector. Samples were shaped into a tablet without any additives. The FT‐IR spectra were measured by the transmission method at around 298 K. The ^27^Al magic‐angle spinning (MAS) NMR spectrum of each montmorillonite was measured by using a nuclear magnetic resonance spectrometer (Bruker AVANCE III 400 WB) with a 4.0‐mm VT‐MAS probe. The equipped rotor is made of ZrO_2_. The sample spinning frequency was 14 kHz. Nitrogen adsorption and desorption measurements at 77 K were performed on a 3FLEX 3500 analyzer (Micromeritics) for samples degassed at 573 K for 3 h. The specific surface area values of the catalysts were determined by using the Brunauer–Emmet–Teller method. Energy‐dispersive X‐ray spectroscopy analysis was performed by using a spectrometer on a scanning transmission electron microscope (JEM‐ARM200F, JEOL) operated at 200 kV.

## Conflict of interest

The authors declare no conflict of interest.

1

## Data Availability

Research data are not shared.
